# Burden of Respiratory Syncytial Virus–Associated Hospitalizations in US Adults, October 2016 to September 2023

**DOI:** 10.1001/jamanetworkopen.2024.44756

**Published:** 2024-11-13

**Authors:** Fiona P. Havers, Michael Whitaker, Michael Melgar, Huong Pham, Shua J. Chai, Elizabeth Austin, James Meek, Kyle P. Openo, Patricia A. Ryan, Chloe Brown, Kathryn Como-Sabetti, Daniel M. Sosin, Grant Barney, Brenda L. Tesini, Melissa Sutton, H. Keipp Talbot, Ryan Chatelain, Pam Daily Kirley, Isaac Armistead, Kimberly Yousey-Hindes, Maya L. Monroe, Val Tellez Nunez, Ruth Lynfield, Chelsea L. Esquibel, Kerianne Engesser, Kevin Popham, Arilene Novak, William Schaffner, Tiffanie M. Markus, Ashley Swain, Monica E. Patton, Lindsay Kim

**Affiliations:** 1 National Center for Immunization and Respiratory Diseases, Centers for Disease Control and Prevention, Atlanta, Georgia; 2 US Public Health Service Commissioned Corps, Rockville, Maryland; 3 California Emerging Infections Program, Oakland; 4 Colorado Department of Public Health and Environment, Denver; 5 Connecticut Emerging Infections Program, Yale School of Public Health, New Haven; 6 Emory University School of Medicine, Atlanta, Georgia; 7 Georgia Emerging Infections Program, Georgia Department of Public Health, Atlanta; 8 Atlanta Veterans Affairs Medical Center, Decatur, Georgia; 9 Maryland Department of Health, Baltimore; 10 Michigan Department of Health and Human Services, Lansing; 11 Minnesota Department of Health, St Paul; 12 New Mexico Department of Health, Santa Fe; 13 New York State Department of Health, Albany; 14 University of Rochester School of Medicine and Dentistry, Rochester, New York; 15 Public Health Division, Oregon Health Authority, Portland; 16 Vanderbilt University Medical Center, Nashville, Tennessee; 17 Salt Lake County Health Department, Salt Lake City, Utah; 18 University of New Mexico Health Sciences Center, Albuquerque

## Abstract

**Importance:**

Respiratory syncytial virus (RSV) infection can cause severe illness in adults. However, there is considerable uncertainty in the burden of RSV-associated hospitalizations among adults prior to RSV vaccine introduction.

**Objective:**

To describe the demographic characteristics of adults hospitalized with laboratory-confirmed RSV and to estimate annual rates and numbers of RSV-associated hospitalizations, intensive care unit (ICU) admissions, and in-hospital deaths.

**Design, Setting, and Participants:**

This cross-sectional study used data from the RSV Hospitalization Surveillance Network (RSV-NET), a population-based surveillance platform that captures RSV-associated hospitalizations in 58 counties in 12 states, covering approximately 8% of the US population. The study period spanned 7 surveillance seasons from 2016-2017 through 2022-2023. Included cases from RSV-NET were nonpregnant hospitalized adults aged 18 years or older residing in the surveillance catchment area and with a positive RSV test result.

**Exposure:**

Laboratory-confirmed RSV-associated hospitalization, defined as a positive RSV test result within 14 days before or during hospitalization.

**Main Outcomes and Measures:**

Hospitalization rates per 100 000 adult population, stratified by age group. After adjusting for test sensitivity and undertesting for RSV in adults hospitalized with acute respiratory illnesses, rates were extrapolated to the US population to estimate annual numbers of RSV-associated hospitalizations. Clinical outcome data were used to estimate RSV-associated ICU admissions and in-hospital deaths.

**Results:**

From the 2016 to 2017 through the 2022 to 2023 RSV seasons, there were 16 575 RSV-associated hospitalizations in adults (median [IQR] age, 70 [58-81] years; 9641 females [58.2%]). Excluding the 2020 to 2021 and the 2021 to 2022 seasons, when the COVID-19 pandemic affected RSV circulation, hospitalization rates ranged from 48.9 (95% CI, 33.4-91.5) per 100 000 adults in 2016 to 2017 to 76.2 (95% CI, 55.2-122.7) per 100 000 adults in 2017 to 2018. Rates were lowest among adults aged 18 to 49 years (8.6 [95% CI, 5.7-16.8] per 100 000 adults in 2016-2017 to 13.1 [95% CI, 11.0-16.1] per 100 000 adults in 2022-2023) and highest among adults 75 years or older (244.7 [95% CI, 207.9-297.3] per 100 000 adults in 2022-2023 to 411.4 [95% CI, 292.1-695.4] per 100 000 adults in 2017-2018). Annual hospitalization estimates ranged from 123 000 (95% CI, 84 000-230 000) in 2016 to 2017 to 193 000 (95% CI, 140 000-311 000) in 2017 to 2018. Annual ICU admission estimates ranged from 24 400 (95% CI, 16 700-44 800) to 34 900 (95% CI, 25 500-55 600) for the same seasons. Estimated annual in-hospital deaths ranged from 4680 (95% CI, 3570-6820) in 2018 to 2019 to 8620 (95% CI, 6220-14 090) in 2017 to 2018. Adults 75 years or older accounted for 45.6% (range, 43.1%-48.8%) of all RSV-associated hospitalizations, 38.6% (range, 36.7%-41.0%) of all ICU admissions, and 58.7% (range, 51.9%-67.1%) of all in-hospital deaths.

**Conclusions and Relevance:**

In this cross-sectional study of adults hospitalized with RSV before the 2023 introduction of RSV vaccines, RSV was associated with substantial burden of hospitalizations, ICU admissions, and in-hospital deaths in adults, with the highest rates occurring in adults 75 years or older. Increasing RSV vaccination of older adults has the potential to reduce associated hospitalizations and severe clinical outcomes.

## Key Points

**Question:**

What was the burden of respiratory syncytial virus (RSV)–associated hospitalizations in US adults prior to the introduction of RSV vaccines?

**Findings:**

In this cross-sectional study using population-based surveillance of 16 575 adults hospitalized with laboratory-confirmed RSV from 2016-2017 through 2022-2023, the annual RSV-associated hospitalization estimates ranged from 123 000 to 193 000, with the highest burden in adults 75 years or older.

**Meaning:**

Findings of this study suggest that before the 2023 introduction of RSV vaccines, RSV was associated with a substantial burden of hospitalizations, intensive care unit admissions, and in-hospital deaths in adults, particularly those 75 years or older.

## Teaser

This cross-sectional study identifies hospitalizations, intensive care unit admissions, and in-hospital deaths associated with respiratory syncytial virus in US adults from 2016 through 2023.

## Introduction

Respiratory syncytial virus (RSV) plays an important role in mortality and morbidity in US adults 60 years or older, although there is considerable uncertainty about the overall burden of RSV-associated hospitalizations in this population.^1,2,3,4,5,6^ As of May 2024, the US Food and Drug Administration had approved 3 vaccines for prevention of RSV lower respiratory tract disease in adults.^7,8,9^ In June 2023, the Centers for Disease Control and Prevention (CDC) recommended a single dose of RSV vaccine in adults aged 60 years or older using shared clinical decision-making^10^; the recommendations were updated in June 2024 to recommend a single dose of RSV vaccine for all adults aged 75 years or older and for those aged 60 to 74 years who are at increased risk for severe RSV disease.^11^ To assess vaccine impact, it is essential to clearly define the prevaccine burden of RSV disease in this population.

Clinicians often do not test for RSV in hospitalized adults with respiratory illness^12^ because an RSV diagnosis does not generally change disease management and because of limited awareness of RSV as an important pathogen affecting adults. Evidence also suggests that standard RSV detection assays in hospitalized adults might have lower sensitivity than previously believed.^13,14,15,16^ Furthermore, potential changes in respiratory virus testing, including during the COVID-19 pandemic, increase uncertainty in estimates of RSV hospitalizations in older adults.^1,2,3,4,5,6^ This analysis used 2016 to 2023 data from the population-based RSV Hospitalization Surveillance Network (RSV-NET) to describe the demographic characteristics of adults 18 years or older hospitalized with laboratory-confirmed RSV and to estimate rates and numbers of RSV-associated hospitalizations, intensive care unit (ICU) admissions, and in-hospital deaths.

## Methods

The Respiratory Virus Hospitalization Surveillance Network (RESP-NET) conducts population-based surveillance for US hospitalizations associated with laboratory-confirmed RSV (recorded in RSV-NET), COVID-19 (recorded in COVID-19 Hospitalization Surveillance Network [COVID-NET]), and influenza (recorded in Influenza Hospitalization Surveillance Network [FluSurv-NET]). During the 2022 to 2023 surveillance season, RSV-NET captured laboratory-confirmed RSV-associated hospitalizations in 58 counties in 12 states (California, Colorado, Connecticut, Georgia, Maryland, Michigan, Minnesota, New Mexico, New York, Oregon, Tennessee, and Utah) covering approximately 27 million persons (approximately 8% of the US population), although the catchment area changed over time (eTable 1 in Supplement 1).^17^ This study was reviewed by the Centers for Disease Control and Prevention (CDC), deemed not research, and conducted consistent with applicable federal law and CDC policy (45 CFR part 46.102(l)(2), 21 CFR part 56; 42 USC §241(d); 5 USC §552a; 44 USC §3501 et seq). We followed the Strengthening the Reporting of Observational Studies in Epidemiology (STROBE) reporting guideline.

Included RSV-NET cases were nonpregnant hospitalized adults aged 18 years or older residing in the surveillance catchment area with a positive molecular or rapid antigen RSV test result during hospitalization or within 14 days before admission. Laboratory testing was ordered at the discretion of clinicians or through hospital screening procedures. Demographic information, including age, sex, race, and Hispanic or Latino ethnicity; hospital admission date; positive RSV test result; and clinical outcomes (ICU admission status and in-hospital deaths) were collected on all patients, allowing calculation of population-based incidence. To characterize the demographic characteristics of adults hospitalized with RSV, race and ethnicity (Hispanic or Latino and non-Hispanic American Indian or Alaska Native, Asian or Pacific Islander, Black, White, or other [including multiracial and unknown]) were abstracted from the medical record.

From 2016 to 2019, prospective surveillance was conducted annually during the typical RSV season, defined as October to April. Beginning in October 2020, surveillance was conducted year-round, and the RSV season was defined as October to September. Incidence was calculated using National Center for Health Statistics vintage 2020 bridged-race postcensal population estimates (2019 to September 2020 and before October 2020) or US Census Bureau vintage unbridged-race postcensal population estimates (October 2020 to September 2023) for surveillance-area counties or county equivalents.^18^

**Figure 1.**
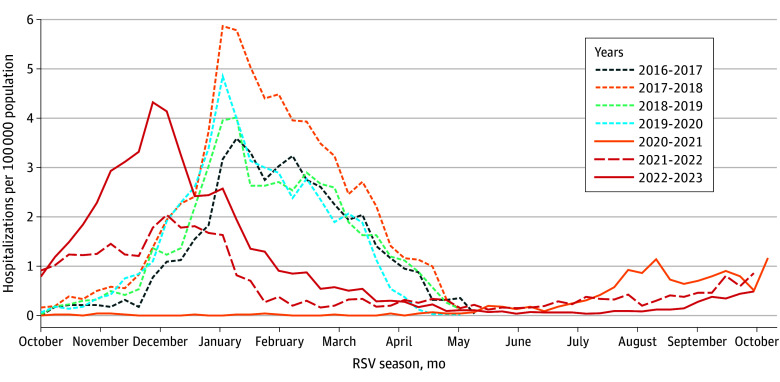
Weekly Adjusted Respiratory Syncytial Virus (RSV)–Associated Hospitalizations per 100 000 Adult Population for the 2016 to 2017 Through 2022 to 2023 Seasons Season is defined as October to April for 2016 to 2017 through 2019 to 2020 and as October to September for 2020 to 2021 through 2022 to 2023.

Adults hospitalized with RSV were captured in RSV-NET only if they were tested for RSV and if the test accurately detected RSV. Because RSV testing is often not performed in hospitalized adults and RSV diagnostic testing practices have changed over time, rates of RSV-associated hospitalizations in RSV-NET were adjusted for underdetection of RSV infection due to testing practices and test sensitivity using a multiplier approach, as previously described for FluSurv-NET.^19,20,21^ Briefly, each RSV-NET site identified all adults hospitalized with acute respiratory illness (ARI), using *International Statistical Classification of Diseases and Related Health Problems, Tenth Revision* (*ICD-10*) diagnosis codes (eTable 2 in Supplement 1), in select hospitals and ascertained the proportion of patients tested for RSV and the type of RSV detection test used among a stratified random sample. Adjustment multipliers, the inverse of the frequency multiplied by the mean sensitivity of the assays, were estimated for each season using testing data from hospitalizations in each age group^22^ (eMethods and eTable 3 in Supplement 1). Unadjusted rates were estimated by dividing the number of laboratory-confirmed hospitalizations detected within the RSV-NET catchment area by the catchment area population estimate. Adjusted rates were estimated by multiplying the unadjusted rates by the adjustment multipliers and then presented with 95% CIs to account for uncertainty in adjustment multipliers. Rates of RSV-associated ICU admissions and in-hospital deaths were estimated by multiplying age-specific hospitalization rates by the proportion of patients in each age group who were admitted to the ICU (ICU to hospitalization ratio) or who died in the hospital^19^ (ratios shown in eTable 3 in Supplement 1). Overall numbers of RSV-associated hospitalizations, ICU admissions, and in-hospital deaths in the US were estimated by multiplying adjusted age-specific rates of hospitalization, ICU admission, and in-hospital death by estimated US populations for each age group.^18^ Starting with the 2020 to 2021 surveillance season, the COVID-19 pandemic affected both RSV circulation and respiratory virus testing practices, affecting RSV-associated hospitalization timing and rates (Figure 1). Demographic characteristics, clinical outcomes, and burden estimates of hospitalizations, ICU admissions, and in-hospital deaths for all seasons are shown in Table 1 and Table 2; 2020 to 2021 and 2021 to 2022 seasons were excluded from these ranges given atypical RSV circulation.

**Table 1.** Demographic Characteristics and Clinical Outcomes of Adults Hospitalized With Laboratory-Confirmed Respiratory Syncytial Virus Infection, 2016 to 2017 Through 2022 to 2023

Characteristic	No. (%) (N = 16 575)^a^
RSV season^a^^,^^b^	
2016-2017	1248 (7.5)
2017-2018	2457 (14.8)
2018-2019	2516 (15.2)
2019-2020	3165 (19.1)
2020-2021	557 (3.4)
2021-2022	2441 (14.7)
2022-2023	4191 (25.3)
Demographics^a^	
Age group, y	
18-49	2230 (13.5)
50-64	3965 (23.9)
≥65	10 380 (62.6)
65-74	3807 (23.0)
≥75	6573 (39.7)
Sex	
Male	6934 (41.8)
Female	9641 (58.2)
Race and ethnicity^c^	
Hispanic or Latino	1429 (8.6)
Non-Hispanic American Indian or Alaska Native	87 (0.5)
Non-Hispanic Asian or Pacific Islander	985 (5.9)
Non-Hispanic Black	3234 (19.5)
Non-Hispanic White	10 187 (61.5)
Other or unknown^d^	653 (3.9)
Clinical outcomes by age group, y^e^	
ICU admission	2844/14 896 (19.1)
18-49	425/2027 (21.0)
50-64	795/3625 (21.9)
65-74	696/3438 (20.2)
≥75	928/5806 (16.0)
In-hospital death	706/16 506 (4.3)
18-49	42/2223 (1.9)
50-64	124/3948 (3.1)
65-74	162/3789 (4.3)
≥75	378/6546 (5.8)

Abbreviations: ICU, intensive care unit; RSV, respiratory syncytial virus.

^a^ For seasons and demographic characteristics, the denominator was 16 575.

^b^ Season was defined as October to April for 2016 to 2017 through 2019 to 2020 and as October to September for 2020 to 2021 through 2022 to 2023.

^c^ Race and ethnicity were abstracted from medical records.

^d^ Other included multiracial. Persons of Hispanic or Latino origin might be of any race but were categorized as Hispanic; all racial groups were categorized as non-Hispanic. All other races included non-Hispanic multiracial persons. If ethnicity was unknown, non-Hispanic ethnicity was assumed.

^e^ For clinical outcomes, the denominators were 14 896 for ICU admission and 16 506 for in-hospital death.

**Table 2.** Estimated Annual Total RSV-Associated Hospitalizations, ICU Admissions, and In-Hospital Deaths by Age Group From 2016 to 2017 Through 2022 to 2023^a^

Age group and RSV season^b^	Estimated No. (95% CI)		
	Hospitalizations	ICU admissions	In-hospital deaths
**18-49 y**			
2016-2017	12 000 (8000-23 000)	2700 (1800-5200)	260 (170-490)
2017-2018	17 000 (13 000-24 000)	3300 (2600-4700)	330 (260-470)
2018-2019	14 000 (11 000-20 000)	3400 (2700-4900)	180 (140-260)
2019-2020	17 000 (15 000-19 000)	3000 (2700-3400)	40 (40-40)
2020-2021	7000 (6000-10 000)	1600 (1400-2300)	170 (140-240)
2021-2022	15 000 (12 000-19 000)	3100 (2500-4000)	340 (270-430)
2022-2023	18 000 (15 000-23 000)	3700 (3100-4700)	540 (450-690)
**50-64 y**			
2016-2017	26 000 (19 000-44 000)	6800 (4900-11 400)	1130 (820-1910)
2017-2018	40 000 (30 000-59 000)	9200 (6900-13 500)	1380 (1040-2040)
2018-2019	28 000 (21 000-40 000)	5500 (4100-7800)	630 (470-900)
2019-2020	28 000 (24 000-33 000)	7400 (6400-8700)	830 (710-980)
2020-2021	9000 (7000-12 000)	1800 (1400-2400)	180 (140-240)
2021-2022	21 000 (18 000-28 000)	3700 (3200-4900)	860 (740-1150)
2022-2023	30 000 (26 000-36 000)	6600 (5700-7900)	830 (720-1000)
**65-74 y**			
2016-2017	25 000 (17 000-43 000)	5700 (3900-9800)	950 (650-1640)
2017-2018	46 000 (33 000-76 000)	9600 (6900-15 800)	2180 (1560-3600)
2018-2019	31 000 (24 000-45 000)	6000 (4700-8800)	1440 (1120-2090)
2019-2020	29 000 (25 000-34 000)	6000 (5100-7000)	1060 (920-1250)
2020-2021	9000 (7000-12 000)	1600 (1200-2100)	490 (380-650)
2021-2022	20 000 (16 000-25 000)	3400 (2700-4300)	670 (540-840)
2022-2023	34 000 (29 000-41 000)	7400 (6300-8900)	1590 (1360-1920)
**≥75 y**			
2016-2017	60 000 (40 000-120 000)	9200 (6100-18 400)	3680 (2450-7360)
2017-2018	90 000 (64 000-152 000)	12 800 (9100-21 600)	4730 (3360-7980)
2018-2019	62 000 (47 000-91 000)	9900 (7500-14 500)	2430 (1840-3570)
2019-2020	58 000 (49 000-70 000)	11 400 (9600-13 700)	3950 (3340-4770)
2020-2021	8000 (7000-12 000)	1100 (900-1600)	480 (420-720)
2021-2022	35 000 (28 000-44 000)	4800 (3800-6000)	1780 (1420-2230)
2022-2023	59 000 (50 000-71 000)	10 000 (8500-12 000)	3960 (3350-4760)
**All ages**			
2016-2017	123 000 (84 000-230 000)	24 400 (16 700-44 800)	6020 (4090-11 400)
2017-2018	193 000 (140 000-311 000)	34 900 (25 500-55 600)	8620 (6220-14 090)
2018-2019	135 000 (103 000-196 000)	24 800 (19 000-36 000)	4680 (3570-6820)
2019-2020	132 000 (113 000-156 000)	27 800 (23 800-32 800)	5880 (5010-7040)
2020-2021	33 000 (27 000-46 000)	6100 (4900-8400)	1320 (1080-1850)
2021-2022	91 000 (74 000-116 000)	15 000 (12 200-19 200)	3650 (2970-4650)
2022-2023	141 000 (120 000-171 000)	27 700 (23 600-33 500)	6920 (5880-8370)
**≥60 y^c^**			
2016-2017	95 000 (64 000-184 000)	17 700 (12 000-33 900)	5310 (3600-10 210)
2017-2018	153 000 (108 000-264 000)	27 200 (19 200-47 100)	7690 (5430-13 260)
2018-2019	105 000 (79 000-157 000)	18 100 (13 700-27 200)	4170 (3140-6220)
2019-2020	100 000 (85 000-121 000)	21 200 (18 000-25 500)	5430 (4600-6560)
2020-2021	21 000 (15 000-30 000)	3500 (2500-4900)	960 (680-1360)
2021-2022	63 000 (51 000-81 000)	9800 (8000-12 700)	2850 (2320-3680)
2022-2023	107 000 (90 000-131 000)	20 400 (17 200-25 000)	5930 (5020-7260)

Abbreviations: ICU, intensive care unit; RSV, respiratory syncytial virus.

^a^ Estimates were adjusted to account for underdetection of RSV infection due to testing practices and test sensitivity.

^b^ Season was defined as October to April for 2016 to 2017 through 2019 to 2020 and as October to September for 2020 to 2021 through 2022 to 2023. Prior to 2020, almost all RSV-associated hospitalizations likely occurred from October through April; seasonal estimates were assumed to approximate annual estimates.

^c^ Estimates for adults aged 60 years or older are shown separately to reflect potential vaccine-avertable RSV–associated hospitalizations, ICU admissions, and in-hospital deaths. In a June 2024 guideline update, RSV vaccination was recommended for all adults aged 75 years or older and for those aged 60 to 74 years who are at an increased risk of severe RSV disease.

Adjustment multipliers for burden estimates accounted for test sensitivity. Recent studies, including a systematic review and meta-analysis, indicated that adding specimen types, such as paired serological testing or sputum to nasopharyngeal or nasal swab reverse transcriptase–polymerase chain reaction (RT-PCR) testing, increased RSV detection by 50% to 66%,^16^ demonstrating that molecular assays with reportedly high sensitivity might underdetect RSV in adults.^13,14,15^ To account for presumed lower sensitivity of PCR testing as performed previously in similar analyses,^2^ we multiplied rate estimates by 1.5, which assumed a PCR testing sensitivity of 66%. We performed a sensitivity analysis using more conservative estimates that did not include the 1.5 multiplier and that presumed a baseline sensitivity of 92%^16^ for molecular assays (PCR) and 29%^23^ for antigen assays.

### Statistical Analysis

Rates were presented per 100 000 adults aged 18 years or older; differences in age group–specific rates were compared using χ^2^ tests. A 2-tailed *P* < .05 was considered to be statistically significant. Data analyses were conducted using SAS, version 9.4 (SAS Institute Inc).

## Results

From the 2016 to 2017 through 2022 to 2023 RSV seasons, 16 575 laboratory-confirmed RSV-associated hospitalizations in adults aged 18 years or older were identified; 98.7% of tests used to detect RSV were RT-PCR. Among hospitalized patients, 9641 were females (58.2%) and 6934 were males (41.8%), with a median (IQR) age of 70 (58-81) years; specifically, 62.6% were 65 years or older and 39.7% were 75 years or older (Table 1; eFigure in Supplement 1). A total of 1429 patients (8.6%) were Hispanic or Latino, and non-Hispanic patients included 87 American Indian or Alaska Native (0.5%), 985 Asian or Pacific Islander (5.9%), 3234 Black (19.5%), and 10 187 White (61.5%) individuals, with 653 (3.9%) having other or unknown race and ethnicity (Table 1).

**Figure 2.**
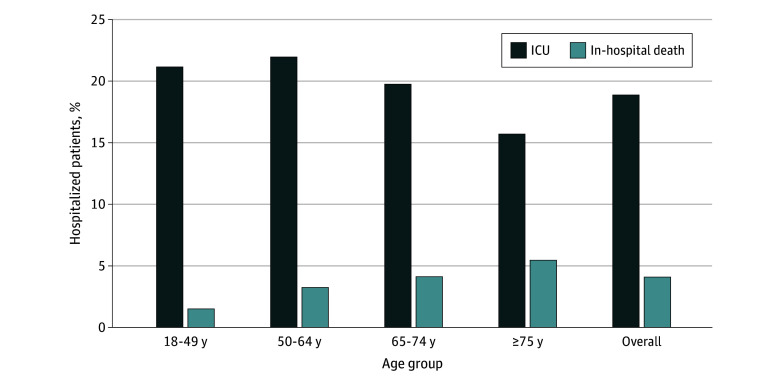
Proportion of Adults With Respiratory Syncytial Virus–Associated Intensive Care Unit (ICU) Stay and In-Hospital Death by Age Group for the 2016 to 2017 Through 2022 to 2023 Seasons Season is defined as October to April for 2016 to 2017 through 2019 to 2020 and as October to September for 2020 to 2021 through 2022 to 2023.

Hospitalization rates peaked each January during 2016 to 2017 through 2019 to 2020, showed reduced and atypical circulation in 2020 to 2021 and 2021 to 2022, and showed increased circulation and an earlier peak in 2022 to 2023 (Figure 1). Across all RSV seasons, 19.1% of patients (2844 of 14 896) were admitted to the ICU, including 21.0% (425 of 2027) of those aged 18 to 49 years; 4.3% (706 of 16 506) died in the hospital, with in-hospital deaths highest among those 75 years or older (5.8% [378 of 6546]) (Figure 2).

**Figure 3.**
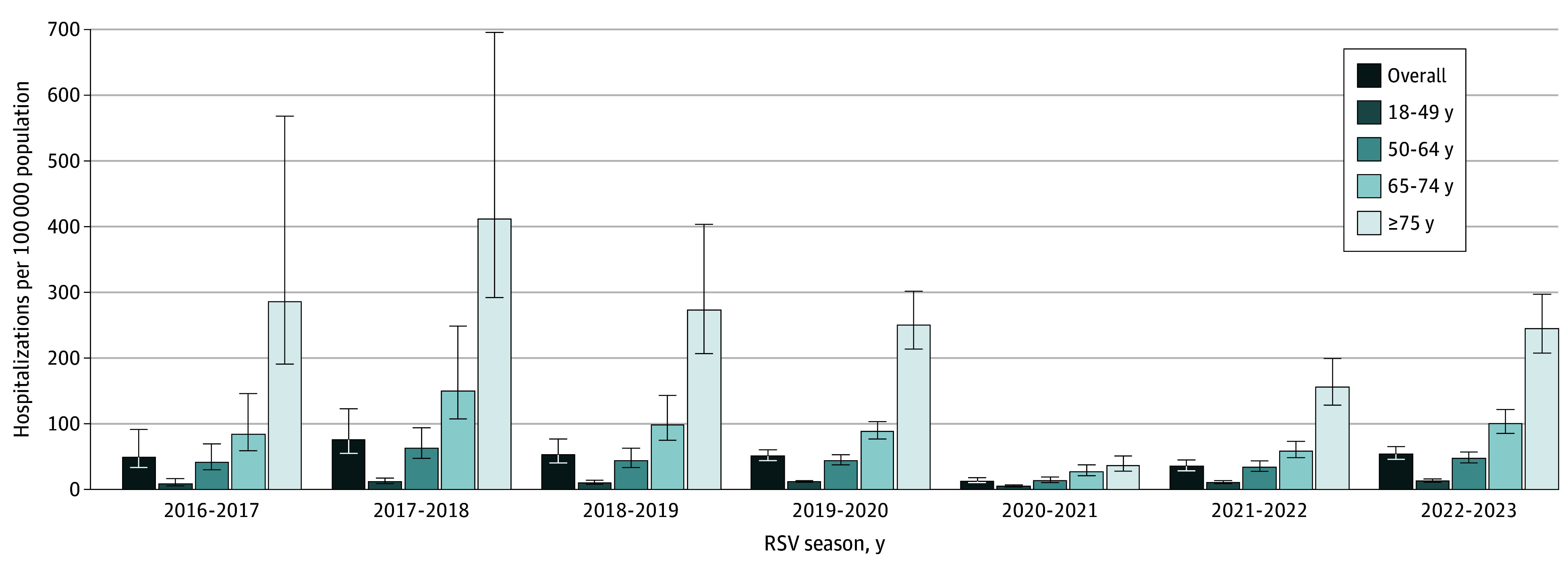
Adjusted Respiratory Syncytial Virus (RSV)–Associated Hospitalizations per 100 000 Adult Population by Age and Season for the 2016 to 2017 Through 2022 to 2023 Seasons Hospitalization rates were adjusted for underdetection of RSV infection due to testing practices and diagnostic test sensitivity. Season is defined as October to April for 2016 to 2017 through 2019 to 2020 and as October to September for 2020 to 2021 through 2022 to 2023. Error bars represent 95% CIs.

Generally, the proportions of adults hospitalized with ARI who were tested for RSV increased over time and were similar across age groups. Across all seasons, 43.5% of hospitalized adults with an ARI were tested for RSV (range, 30.0%-60.7%). In 2016 to 2017, 30.4% of those aged 18 to 49 years, 33.1% of those aged 50 to 64 years, 31.5% of those aged 65 to 74 years, and 27.7% of those 75 years or older were tested. In 2022 to 2023, testing increased to 56.1%, 61.2%, 62.3%, and 61.6% in the same age groups, respectively (eTable 3 in Supplement 1). Unadjusted overall rates of laboratory-confirmed hospitalizations within the RSV-NET catchment area ranged from 8.8 per 100 000 adults in 2016 to 2017 to 21.2 per 100 000 adults in 2022 to 2023. Using adjustment multipliers to account for the proportion tested and test sensitivity (range of 3.2-5.4, by season and age group), seasonal hospitalization rates for adults 18 years or older ranged from 48.9 (95% CI, 33.4-91.5) per 100 000 adults in 2016 to 2017 to 76.2 (95% CI, 55.2-122.7) per 100 000 adults in 2017 to 2018 (Figure 3; eTable 3 in Supplement 1). Results from the sensitivity analysis where a higher RT-PCR sensitivity (92%) was assumed (ie, without the 1.5 × adjustment multiplier) are provided in eTable 4 in Supplement 1.

Hospitalization rates increased with increasing age (Figure 3; eTable 5 in Supplement 1). Adjusted rates were lowest in those aged 18 to 49 years, varying from 8.6 (95% CI, 5.7-16.8) per 100 000 adults in 2016 to 2017 to 13.1 (95% CI, 11.0-16.1) per 100 000 adults in 2022 to 2023 (eTable 3 in Supplement 1). Adjusted rates were highest among those aged 75 years or older, varying from 244.7 (95% CI, 207.9-297.3) per 100 000 adults in 2022 to 2023 to 411.4 (95% CI, 292.1-695.4) per 100 000 adults in 2017 to 2018.

Extrapolating adjusted rates to the US adult population, estimated numbers of annual RSV-associated hospitalizations in adults aged 18 years or older ranged from 123 000 (95% CI, 84 000-230 000) in 2016 to 2017 to 193 000 (95% CI, 140 000-311 000) in 2017 to 2018 (Table 2), while annual ICU admission estimates ranged from 24 400 (95% CI, 16 700-44 800) to 34 900 (95% CI, 25 500-55 600) for the same seasons. Estimated annual in-hospital deaths ranged from 4680 (95% CI, 3570-6820) in 2018 to 2019 to 8620 (95% CI, 6220-14 090) in 2017 to 2018 (Table 2). Averaging across seasons, most adult disease burden occurred in adults aged 65 years or older, including 68.2% (range, 65.9%-70.4%) of all hospitalizations, 63.1% (range, 62.5%-64.2%) of ICU admissions, and 81.1% (range, 76.9%-83.0%) of in-hospital deaths. Estimated numbers of hospitalizations in adults 65 years or older ranged from 85 000 (95% CI, 57 000-163 000) in 2016 to 2017 to 136 000 (95% CI, 97 000-228 000) in 2017 to 2018 (eTable 5 in Supplement 1). Averaging across seasons, adults aged 75 years or older accounted for 45.6% (range, 43.1%-48.8%) of all hospitalizations, 38.6% (range, 36.7%-41.0%) of all ICU admissions, and 58.7% (range, 51.9%-67.1%) of all in-hospital deaths.

## Discussion

Analysis of data on 16 575 laboratory-confirmed RSV-associated hospitalizations in US adults across multiple seasons obtained from RSV-NET, a large, population-based, geographically diverse surveillance platform, showed estimated RSV-associated hospitalization rates of 48.9 to 76.2 per 100 000 adults per season. These rates suggest a high burden of severe RSV disease, with an estimated 123 000 to 193 000 hospitalizations, 24 400 to 34 900 ICU admissions, and 4680 to 8620 in-hospital deaths occurring annually among US adults, although there was a considerable amount of uncertainty surrounding these estimates. Almost half of all hospitalizations and most deaths occurred in adults aged 75 years or older. RSV-associated hospitalizations resulted in considerable morbidity and mortality; nearly 20% of hospitalized patients were admitted to the ICU, and approximately 1 in 25 died in the hospital.

These findings validated RSV as a substantial contributor to respiratory illness and hospitalization among adults, especially older adults, with up to 136 000 estimated annual hospitalizations among those 65 years or older. The burden of RSV-associated hospitalizations in older adults was comparable to the burden of influenza-associated hospitalizations during milder influenza seasons; notwithstanding reductions in influenza disease achieved through vaccination, 87 000 to 523 000 hospitalizations in adults 65 years or older^20^ were estimated to occur annually based on data from FluSurv-NET, an RESP-NET network with methods and catchment area nearly identical to those of RSV-NET. Similarly, RSV disease severity among hospitalized adults appeared to be comparable to or possibly more than the severity of influenza and SARS-CoV-2 in hospitalized adults.^5,24,25,26,27^ In the present analysis, 19.1% of adults hospitalized with RSV were admitted to the ICU and 4.3% died in the hospital, comparable to study of an RESP-NET analysis of adults hospitalized in 2021 to 2022 that found among those with laboratory-confirmed SARS-CoV-2, 15.5% were admitted to the ICU and 4.6% died; for influenza, those proportions were 13.3% and 4.6%, respectively.^25^ These proportions of patients with RSV who were admitted to the ICU and died in the hospital were comparable to those in other studies of RSV,^4,26,28,29,30^ including the 7.1% in-hospital case-fatality proportion estimated for adults aged 60 years or older in a systematic review and meta-analysis of data from high-income countries.^31^ Even among patients aged 18 to 49 years who were hospitalized with RSV, clinical outcomes were severe; in the present study, 21.0% were admitted to the ICU, likely reflecting the high proportion of younger adult patients hospitalized with RSV who were immunocompromised or had underlying medical conditions that made them vulnerable to severe disease.^32^ In-hospital deaths occurred in over 4.0% of adults aged 18 years or older in our analysis, which was likely an underestimation of RSV mortality given that a substantial proportion of deaths may occur after hospital discharge.^29,33^

The wide CIs for estimated hospitalization rates based on RSV-NET data reflect considerable uncertainty in estimating the true burden of RSV-associated hospitalizations in the US. This uncertainty is exacerbated by the suboptimal sensitivity of available RSV detection tests and lack of testing for RSV in adults hospitalized with respiratory illnesses, as demonstrated by less than half of adults hospitalized with ARI in participating RSV-NET hospitals being tested for RSV. Despite the uncertainty in these estimates, RSV-NET–based hospitalization rate and burden estimates are comparable to other burden estimates, including an industry-sponsored meta-analysis and systematic review estimating that RSV was associated with 108 834 hospitalizations and 7763 deaths in 2019 in adults 60 years or older.^31^ Another industry-sponsored systematic review and meta-analysis, which similarly adjusted for underdetection of RSV by nasopharyngeal or nasal RT-PCR alone (eg, applied the RSV detection multiplier of 1.5) and used pooled estimates of hospitalization rates from prospective^1,4,5,6,34^ and modeling studies,^35,36,37,38^ estimated that 17 700, 42 060, and 159 247 RSV-associated hospitalizations occurred annually in adults aged 18 to 49 years, 50 to 64 years, and 65 years or older, respectively,^2^ which were higher than RSV-NET–based estimates but were within estimated CIs for most seasons. Five prospective studies were the source of the estimates, although several studies were limited to a single season^4^ or a restricted geographic area,^1,4,5,6,34^ which might have affected rate estimates. Unlike these studies, RSV-NET uses population-based surveillance in multiple sites in geographically diverse areas.

Although RSV is now a vaccine-preventable disease,^39^ its role in acute respiratory disease continues to be underrecognized by clinicians specializing in adult care.^40^ In the present analysis, less than half of the patients hospitalized with ARI were tested for RSV, consistent with other literature,^12^ although the proportion of patients who were tested for RSV increased from 2016 to 2017 through 2022 to 2023 in all age groups. The availability of vaccines and the increased use of multiplex respiratory virus testing might increase clinicians’ and patients’ awareness of RSV as a cause of illness in adults. This analysis also highlighted the potential public health impact of RSV vaccination among older adults. RSV particularly affects adults aged 75 years or older, a finding consistently reported by studies estimating RSV-associated hospitalization disease burden.^1,2,31^ RSV vaccination is effective in preventing hospitalization in adults aged 60 years or older^41^ and is recommended in the US for adults aged 75 years or older and those aged 60 to 74 years who are at an increased risk for severe RSV disease.^11^ Early vaccination coverage estimates indicated that in the US, less than 20% of adults aged 60 years or older and less than 10% of nursing home residents had received RSV vaccination by December 2023.^42,43,44^ Reducing barriers to vaccination and increasing clinician awareness of the disease burden associated with RSV are needed to increase use of RSV vaccines. Additional population-based epidemiologic analyses are planned to examine risk factors beyond patient age, such as underlying medical conditions, race and ethnicity, rural residence, and social determinants of health, that identify individuals at greatest risk for severe clinical outcomes.

The data we collected and analyzed also demonstrated how the COVID-19 pandemic disrupted the consistent prepandemic seasonality of RSV-associated hospitalizations, with 2 years of atypical seasonality and lower circulation, followed by a surge of RSV-associated hospitalizations in the fall of 2022 with an earlier peak than in prepandemic seasons. This disruption of RSV circulation has been well documented in other studies,^45,46,47^ likely because of nonpharmaceutical interventions such as mask wearing, school closure, and social distancing. These data, along with data from FluSurv-NET and COVID-NET, demonstrate the importance of ongoing, robust, population-based hospital surveillance to track major changes in the burden and epidemiologic pattern of seasonal respiratory viruses.

### Limitations

There are multiple limitations to this analysis. First, decisions about RSV testing were made at the discretion of the treating clinician. Estimates of RSV burden based only on laboratory-confirmed hospitalizations underestimate the true burden of disease in adults. Second, although adjustments for underdetection of RSV were made related to testing practices and test sensitivity, assumptions underlying adjustment multipliers may not be correct. These assumptions included that poor test sensitivity was random and that patients hospitalized with ARI who were not tested for RSV were as likely to have RSV as patients who were tested; if untested patients were less likely to have RSV infection, then these methods may overestimate the burden of disease. In contrast, these adjustments did not account for hospitalization of adults with nonrespiratory complications of RSV (eg, heart failure exacerbation)^48^ who were not tested for RSV and who may not have an assigned *ICD-10* diagnosis code for ARI, potentially leading to an underestimation of RSV-attributable hospitalizations.

Third, this study did not evaluate reasons for admission, and RSV may not be the primary reason for hospitalization. However, previous RSV-NET–based analyses have shown that most hospitalized adults with an RSV diagnosis experience substantial respiratory illness, suggesting that mild or asymptomatic RSV infections make up only a small percentage of detected RSV-associated hospitalizations.^48,49^ Fourth, patients with more severe illness might have been more likely to be tested for RSV, resulting in a potential overestimation of the proportion of patients with RSV-associated hospitalizations who were admitted to the ICU or who died. Fifth, the demographic characteristics of people in the RSV-NET surveillance area were generally similar to those of the US population. However, RSV-NET data might not be generalizable to the entire country, and the methods in this study might not adequately account for the uncertainty in extrapolating RSV-NET data nationally.

## Conclusions

This cross-sectional study of adults hospitalized with laboratory-confirmed RSV demonstrated that prior to the availability of vaccines in 2023, RSV was associated with a substantial burden of disease in adults, particularly older adults. In the US, effective RSV vaccines^27^ have become available and vaccination is now recommended for all adults aged 75 years or older and for those aged 60 to 74 years who are at an increased risk for severe RSV disease. The study found that most hospitalizations occurred among older adults, with the highest hospitalization rates in those aged 75 years or older. Given the large numbers of potentially vaccine-preventable hospitalizations and deaths associated with RSV, increasing vaccine coverage among adults at highest risk could reduce associated hospitalizations and severe clinical outcomes.

## Notes

Supplement 1. **eTable 1.** Changes in the RSV-NET Surveillance Population From 2016 to 2017 Season Through 2022 to 2023 Season **eTable 2.** ICD-10 Codes Used to Identify Patients Hospitalized With Acute Respiratory Illnesses **eTable 3.** Estimated Multipliers for Under-Detection of Respiratory Syncytial Virus Among Hospitalized Adults in RSV-NET, 2016–17 Through 2022–23 **eTable 4.** Estimated Rates and Number of Respiratory Syncytial Virus Hospitalizations in the US by Age Group and Season Assuming a Higher Diagnostic Test Sensitivity (92%) to Account for Under-Detection by Nasopharyngeal Swabs **eTable 5.** Adjusted Hospitalization Rates, Estimated Hospitalizations, Intensive Care Unit (ICU) Admissions and In-Hospital Deaths With Rates Shown in 5-Year Age Bands for Adults ≥50 Years and for All Adults ≥65 Years **eFigure.** Proportion of Adults Hospitalized With Respiratory Syncytial Virus by Age Group and Season
eMethods.

eReferences.

Supplement 2.
Data Sharing Statement

## References

[1] Branche AR, Saiman L, Walsh EE, . Incidence of respiratory syncytial virus infection among hospitalized adults, 2017-2020. Clin Infect Dis. 2022;74(6):1004-1011. doi:10.1093/cid/ciab595 34244735

[2] McLaughlin JM, Khan F, Begier E, Swerdlow DL, Jodar L, Falsey AR. Rates of medically attended RSV among US adults: a systematic review and meta-analysis. Open Forum Infect Dis. 2022;9(7):ofac300. doi:10.1093/ofid/ofac300 35873302 PMC9301578

[3] Falsey AR. Respiratory syncytial virus infection in elderly and high-risk adults. Exp Lung Res. 2005;31(suppl 1):77. doi:10.1056/NEJMoa043951 16395866

[4] Widmer K, Griffin MR, Zhu Y, Williams JV, Talbot HK. Respiratory syncytial virus- and human metapneumovirus-associated emergency department and hospital burden in adults. Influenza Other Respir Viruses. 2014;8(3):347-352. doi:10.1111/irv.12234 24512531 PMC3984605

[5] Widmer K, Zhu Y, Williams JV, Griffin MR, Edwards KM, Talbot HK. Rates of hospitalizations for respiratory syncytial virus, human metapneumovirus, and influenza virus in older adults. J Infect Dis. 2012;206(1):56-62. doi:10.1093/infdis/jis309 22529314 PMC3415933

[6] Belongia EA, King JP, Kieke BA, . Clinical features, severity, and incidence of RSV illness during 12 consecutive seasons in a community cohort of adults ≥60 years old. Open Forum Infect Dis. 2018;5(12):ofy316. doi:10.1093/ofid/ofy316 30619907 PMC6306566

[7] Abrysvo. Package insert. US Food and Drug Administration. Accessed September 11, 2024. https://www.fda.gov/media/168889/download?attachment

[8] Arexvy. Package insert. US Food and Drug Administration. Accessed September 11, 2024. https://www.fda.gov/media/167805/download?attachment

[9] Mresvia. Package insert. US Food and Drug Administration. Accessed September 11, 2024. https://www.fda.gov/media/179005/download

[10] Melgar M, Britton A, Roper LE, . Use of respiratory syncytial virus vaccines in older adults: recommendations of the Advisory Committee on Immunization Practices—United States, 2023. MMWR Morb Mortal Wkly Rep. 2023;72(29):793-801. doi:10.15585/mmwr.mm7229a4 37471262 PMC10360650

[11] Britton A, Roper LE, Kotton CN, . Use of respiratory syncytial virus vaccines in adults aged ≥60 years: updated recommendations of the Advisory Committee on Immunization Practices—United States, 2024. MMWR Morb Mortal Wkly Rep. 2024;73(32):696-702. doi:10.15585/mmwr.mm7332e1 39146277

[12] Rozenbaum MH, Judy J, Tran D, Yacisin K, Kurosky SK, Begier E. Low levels of RSV testing among adults hospitalized for lower respiratory tract infection in the United States. Infect Dis Ther. 2023;12(2):677-685. doi:10.1007/s40121-023-00758-5 36707466 PMC9883084

[13] Zhang Y, Sakthivel SK, Bramley A, . Serology enhances molecular diagnosis of respiratory virus infections other than influenza in children and adults hospitalized with community-acquired pneumonia. J Clin Microbiol. 2016;55(1):79-89. doi:10.1128/JCM.01701-16 27795341 PMC5228265

[14] Bouzid D, Hingrat QL, Salipante F, . Agreement of respiratory viruses’ detection between nasopharyngeal swab and bronchoalveolar lavage in adults admitted for pneumonia: a retrospective study. Clin Microbiol Infect. 2023;29(7):942.e1-942.e6. doi:10.1016/j.cmi.2022.12.024 36708772 PMC9873593

[15] Ramirez J, Carrico R, Wilde A, . Diagnosis of respiratory syncytial virus in adults substantially increases when adding sputum, saliva, and serology testing to nasopharyngeal swab RT-PCR. Infect Dis Ther. 2023;12(6):1593-1603. doi:10.1007/s40121-023-00805-1 37148463 PMC10163290

[16] Onwuchekwa C, Moreo LM, Menon S, . Under-ascertainment of respiratory syncytial virus infection in adults due to diagnostic testing limitations: a systematic literature review and meta-analysis. J Infect Dis. 2023;228(2):173-184. doi:10.1093/infdis/jiad012 36661222 PMC10345483

[17] RSV-NET. Centers for Disease Control and Prevention. Accessed September 6, 2024. https://www.cdc.gov/rsv/php/surveillance/rsv-net.html

[18] U.S. Census populations with bridged race categories. Centers for Disease Control and Prevention. Accessed August 4, 2021. https://www.cdc.gov/nchs/nvss/bridged_race.htm

[19] Reed C, Chaves SS, Daily Kirley P, . Estimating influenza disease burden from population-based surveillance data in the United States. PLoS One. 2015;10(3):e0118369. doi:10.1371/journal.pone.0118369 25738736 PMC4349859

[20] Rolfes MA, Foppa IM, Garg S, . Annual estimates of the burden of seasonal influenza in the United States: a tool for strengthening influenza surveillance and preparedness. Influenza Other Respir Viruses. 2018;12(1):132-137. doi:10.1111/irv.12486 29446233 PMC5818346

[21] Kamidani S, Garg S, Rolfes MA, . Epidemiology, clinical characteristics, and outcomes of influenza-associated hospitalizations in US children over 9 seasons following the 2009 H1N1 pandemic. Clin Infect Dis. 2022;75(11):1930-1939. doi:10.1093/cid/ciac296 35438769

[22] Millman AJ, Reed C, Kirley PD, . Improving accuracy of influenza-associated hospitalization rate estimates. Emerg Infect Dis. 2015;21(9):1595-1601. doi:10.3201/eid2109.141665 26292017 PMC4550134

[23] Chartrand C, Tremblay N, Renaud C, Papenburg J. Diagnostic accuracy of rapid antigen detection tests for respiratory syncytial virus infection: systematic review and meta-analysis. J Clin Microbiol. 2015;53(12):3738-3749. doi:10.1128/JCM.01816-15 26354816 PMC4652120

[24] Surie D, Yuengling KA, DeCuir J, ; IVY Network. Disease severity of respiratory syncytial virus compared with COVID-19 and influenza among hospitalized adults aged ≥60 years—IVY Network, 20 U.S. states, February 2022-May 2023. MMWR Morb Mortal Wkly Rep. 2023;72(40):1083-1088. doi:10.15585/mmwr.mm7240a2 37796753 PMC10564326

[25] Kojima N, Taylor CA, Tenforde MW, . Clinical outcomes of US adults hospitalized for COVID-19 and influenza in the Respiratory Virus Hospitalization Surveillance Network, October 2021-September 2022. Open Forum Infect Dis. 2023;11(1):ofad702. doi:10.1093/ofid/ofad702 38269052 PMC10807992

[26] Begley KM, Monto AS, Lamerato LE, . Prevalence and clinical outcomes of respiratory syncytial virus vs influenza in adults hospitalized with acute respiratory illness from a prospective multicenter study. Clin Infect Dis. 2023;76(11):1980-1988. doi:10.1093/cid/ciad031 36694363 PMC10250013

[27] Surie D, Yuengling KA, DeCuir J, ; Investigating Respiratory Viruses in the Acutely Ill (IVY) Network. Severity of respiratory syncytial virus vs COVID-19 and influenza among hospitalized US adults. JAMA Netw Open. 2024;7(4):e244954. doi:10.1001/jamanetworkopen.2024.4954 38573635 PMC11192181

[28] Kujawski SA, Whitaker M, Ritchey MD, . Rates of respiratory syncytial virus (RSV)-associated hospitalization among adults with congestive heart failure—United States, 2015-2017. PLoS One. 2022;17(3):e0264890. doi:10.1371/journal.pone.0264890 35263382 PMC8906631

[29] Tseng HF, Sy LS, Ackerson B, . Severe morbidity and short- and mid- to long-term mortality in older adults hospitalized with respiratory syncytial virus infection. J Infect Dis. 2020;222(8):1298-1310. doi:10.1093/infdis/jiaa361 32591787

[30] Pastula ST, Hackett J, Coalson J, . Hospitalizations for respiratory syncytial virus among adults in the United States, 1997-2012. Open Forum Infect Dis. 2017;4(1):ofw270. doi:10.1093/ofid/ofw270 28480262 PMC5414053

[31] Savic M, Penders Y, Shi T, Branche A, Pircon JY. Respiratory syncytial virus disease burden in adults aged 60 years and older in high-income countries: a systematic literature review and meta-analysis. Influenza Other Respir Viruses. 2022;17(1):e13031. doi:10.1111/irv.13031 36369772 PMC9835463

[32] Nam HH, Ison MG. Respiratory syncytial virus infection in adults. BMJ. 2019;366:l5021. doi:10.1136/bmj.l5021 31506273

[33] Branche AR, Saiman L, Walsh EE, . Change in functional status associated with respiratory syncytial virus infection in hospitalized older adults. Influenza Other Respir Viruses. 2022;16(6):1151-1160. doi:10.1111/irv.13043 36069297 PMC9530534

[34] McClure DL, Kieke BA, Sundaram ME, . Seasonal incidence of medically attended respiratory syncytial virus infection in a community cohort of adults ≥50 years old. PLoS One. 2014;9(7):e102586. doi:10.1371/journal.pone.0102586 25025344 PMC4099308

[35] Matias G, Taylor R, Haguinet F, Schuck-Paim C, Lustig R, Shinde V. Estimates of hospitalization attributable to influenza and RSV in the US during 1997-2009, by age and risk status. BMC Public Health. 2017;17(1):271. doi:10.1186/s12889-017-4177-z 28320361 PMC5359836

[36] Goldstein E, Greene SK, Olson DR, Hanage WP, Lipsitch M. Estimating the hospitalization burden associated with influenza and respiratory syncytial virus in New York City, 2003-2011. Influenza Other Respir Viruses. 2015;9(5):225-233. doi:10.1111/irv.12325 25980600 PMC4548992

[37] Mullooly JP, Bridges CB, Thompson WW, ; Vaccine Safety Datalink Adult Working Group. Influenza- and RSV-associated hospitalizations among adults. Vaccine. 2007;25(5):846-855. doi:10.1016/j.vaccine.2006.09.041 17074423

[38] Zhou H, Thompson WW, Viboud CG, . Hospitalizations associated with influenza and respiratory syncytial virus in the United States, 1993-2008. Clin Infect Dis. 2012;54(10):1427-1436. doi:10.1093/cid/cis211 22495079 PMC3334364

[39] Scruggs-Wodkowski EA, Malani PN, Linder KA. Therapies to decrease severe respiratory syncytial virus illness. JAMA. 2024;331(24):2127-2128. doi:10.1001/jama.2024.7406 38814625

[40] Hurley LP, Allison MA, Kim L, . Primary care physicians’ perspectives on respiratory syncytial virus (RSV) disease in adults and a potential RSV vaccine for adults. Vaccine. 2019;37(4):565-570. doi:10.1016/j.vaccine.2018.12.031 30598385

[41] Surie D, Self WH, Zhu Y, ; Investigating Respiratory Viruses in the Acutely Ill (IVY) Network. RSV vaccine effectiveness against hospitalization among US adults 60 years and older. JAMA. 2024;332(13):1105-1107. doi:10.1001/jama.2024.15775 39230920 PMC11375516

[42] Black CL, Kriss JL, Razzaghi H, . Influenza, updated COVID-19, and respiratory syncytial virus vaccination coverage among adults—United States, Fall 2023. MMWR Morb Mortal Wkly Rep. 2023;72(51):1377-1382. doi:10.15585/mmwr.mm7251a4 38127675 PMC10754266

[43] Reses HE, Dubendris H, Haas L, . Coverage with influenza, respiratory syncytial virus, and updated COVID-19 vaccines among nursing home residents—National Healthcare Safety Network, United States, December 2023. MMWR Morb Mortal Wkly Rep. 2023;72(51):1371-1376. doi:10.15585/mmwr.mm7251a3 38127673 PMC10754267

[44] Centers for Disease Control and Prevention. National Immunization Survey-Adult COVID Module (NIS-ACM). Accessed September 11, 2024. https://www.cdc.gov/rsvvaxview/dashboard/adults-60-coverage-intent.html?CDC_AAref_Val=https://www.cdc.gov/vaccines/imz-managers/coverage/rsvvaxview/adults-60-coverage-intent.html

[45] Falsey AR, Cameron A, Branche AR, Walsh EE. Perturbations in respiratory syncytial virus activity during the SARS-CoV-2 pandemic. J Infect Dis. 2022;227(1):83-86. doi:10.1093/infdis/jiac434 36315855

[46] Munkstrup C, Lomholt FK, Emborg HD, . Early and intense epidemic of respiratory syncytial virus (RSV) in Denmark, August to December 2022. Euro Surveill. 2023;28(1):2200937. doi:10.2807/1560-7917.ES.2023.28.1.2200937 36695451 PMC9817209

[47] Mrcela D, Markic J, Zhao C, . Changes following the onset of the COVID-19 pandemic in the burden of hospitalization for respiratory syncytial virus acute lower respiratory infection in children under two years: a retrospective study from Croatia. Viruses. 2022;14(12):2746. doi:10.3390/v14122746 36560751 PMC9785187

[48] Woodruff RC, Melgar M, Pham H, ; Respiratory Syncytial Virus Hospitalization Surveillance Network (RSV-NET). Acute cardiac events in hospitalized older adults with respiratory syncytial virus infection. JAMA Intern Med. 2024;184(6):602-611. doi:10.1001/jamainternmed.2024.0212 38619857 PMC11019447

[49] Havers FP, Whitaker M, Melgar M, ; RSV-NET Surveillance Team. Characteristics and outcomes among adults aged ≥60 years hospitalized with laboratory-confirmed respiratory syncytial virus—RSV-NET, 12 states, July 2022-June 2023. MMWR Morb Mortal Wkly Rep. 2023;72(40):1075-1082. doi:10.15585/mmwr.mm7240a1 37796742 PMC10564327

