# Supplementary material for: Burden of Respiratory Syncytial Virus–Associated Hospitalizations in US Adults, October 2016 to September 2023
Source: JAMA Netw Open. 2024 Nov 13;7(11):e2444756. doi: 10.1001/jamanetworkopen.2024.44756 (PMC11561688; doi:10.1001/jamanetworkopen.2024.44756)
Supplement: Supplement 1. — eTable 1. Changes in the RSV-NET Surveillance Population From 2016 to 2017 Season Through 2022 to 2023 Season eTable 2. ICD-10 Codes Used to Identify Patients Hospitalized With Acute Respiratory Illnesses eTable 3. Estimated Multipliers for Under-Detection of Respiratory Syncytial Virus Among Hospitalized Adults in RSV-NET, 2016–17 Through 2022–23 eTable 4. Estimated Rates and Number of Respiratory Syncytial Virus Hospitalizations in the US by Age Group and Season Assuming a Higher Diagnostic Test Sensitivity (92%) to Account for Under-Detection by Nasopharyngeal Swabs eTable 5. Adjusted Hospitalization Rates, Estimated Hospitalizations, Intensive Care Unit (ICU) Admissions and In-Hospital Deaths With Rates Shown in 5-Year Age Bands for Adults ≥50 Years and for All Adults ≥65 Years eFigure. Proportion of Adults Hospitalized With Respiratory Syncytial Virus by Age Group and Season eMethods. eReferences. [file jamanetwopen-e2444756-s001.pdf]

Supplemental Online Content

Havers FP, Whitaker M, Melgar M, et al. Burden of respiratory syncytial virus–associated hospitalizations in US adults, October 2016 to September 2023. *JAMA Netw Open*. 2024;7(11):e2444756. doi:10.1001/jamanetworkopen.2024.44756

eTable 1. Changes in the RSV-NET Surveillance Population From 2016 to 2017 Season Through 2022 to 2023 Season

eTable 2. ICD-10 Codes Used to Identify Patients Hospitalized With Acute Respiratory Illnesses

eTable 3. Estimated Multipliers for Under-Detection of Respiratory Syncytial Virus Among Hospitalized Adults in RSV-NET, 2016–17 Through 2022–23

eTable 4. Estimated Rates and Number of Respiratory Syncytial Virus Hospitalizations in the U.S. by Age Group and Season Assuming a Higher Diagnostic Test Sensitivity (92%) to Account for Under-Detection by Nasopharyngeal Swabs

eTable 5. Adjusted Hospitalization Rates, Estimated Hospitalizations, Intensive Care Unit (ICU) Admissions and In-Hospital Deaths With Rates Shown in 5-Year Age Bands for Adults ≥50 Years and for All Adults ≥65 Years

eFigure. Proportion of Adults Hospitalized With Respiratory Syncytial Virus by Age Group and Season

eMethods.

eReferences.

This supplemental material has been provided by the authors to give readers additional information about their work.

© 2024 Havers FP et al. *JAMA Network Open*

eTable 1. Changes in the RSV-NET Surveillance Population From 2016 to 2017 Season Through 2022 to 2023 Season

Season	Surveillance Period	Surveillance Population
2016–2017	October 2016 – April 2017	Adults in CA, GA, MD, MN, NY, OR, TN
2017–2018	October 2017 – April 2018	Adults in CA, GA, MD, MN, NM, NY, OR, TN
2018–2019	October 2018 – April 2019	Adults in all participating states*
2019–2020	October 2019 – April 2020	Adults in all participating states*
2020–2021	October 2020 – September 2021	Adults in all participating states*
2021–2022	October 2021 – September 2022	Adults in all participating states*
2022-2023	October 2023 – September 2023	Adults in all participating states*

*The 12 participating states are California, Colorado, Connecticut, Georgia, Maryland, Michigan, Minnesota, New Mexico, New York, Oregon, Tennessee, and Utah. The RSV-NET catchment area includes select counties in these states.

© 2024 Havers FP et al. *JAMA Network Open*

eTable 2. ICD-10 codes used to identify hospital admissions for acute respiratory illnesses.

ICD-10 Code	ICD-10 Code Description
**J00**	Acute nasopharyngitis (common cold)
**J01.***	Acute sinusitis
**J02.***	Acute pharyngitis
**J03.***	Acute tonsillitis
**J04.***	Acute laryngitis and tracheitis
**J05.***	Acute obstructive laryngitis and epiglottis
**J06.***	Acute upper respiratory infection of multiple and unspecified sites
**J09.***	Influenza due to certain identified influenza viruses
**J10.***	Influenza due to other identified influenza virus
**J11.***	Influenza due to unidentified influenza virus
**J12**	Viral pneumonia, not elsewhere classified
**J12.0**	Adenoviral pneumonia
**J12.1**	Respiratory syncytial virus pneumonia
**J12.2**	Parainfluenza virus pneumonia
**J12.3**	Human metapneumovirus pneumonia
**J12.8**	Other viral pneumonia
**J12.81**	Pneumonia due to SARS-associated coronavirus
**J12.82**	Pneumonia due to coronavirus disease 2019
**J12.89**	Other viral pneumonia, influenza bronchopneumonia
**J12.9**	Viral pneumonia, unspecified
**J13**	*Streptococcus pneumoniae* Pneumonia
**J14**	*Hemophilus influenzae* Pneumonia
**J15**	Bacterial pneumonia not elsewhere classified
**J15.0**	*Klebsiella pneumoniae* Pneumonia
**J15.1**	Pneumonia due to *Pseudomonas*
**J15.2**	Pneumonia due to *Staphylococcus*
**J15.20**	*Staphylococcus*, unspecified
**J15.211**	MSSA Pneumonia
**J15.212**	MRSA Pneumonia
**J15.29**	Other *Staphylococcus* pneumonia
**J15.3**	*Streptococcus, group B*
**J15.4**	Pneumonia due to other *Streptococcus*
**J15.5**	Pneumonia due to *Escherichia coli*
**J15.6**	Other gram-negative bacteria
**J15.7**	Pneumonia due to *Mycoplasma pneumoniae*
**J15.8**	Pneumonia due to other specified bacteria
**J15.9**	Bacterial pneumonia, unspecified

© 2024 Havers FP et al. *JAMA Network Open*

**J16.***	Pneumonia due to other specified infectious organism, not elsewhere classified
**J17**	Pneumonia in diseases classified elsewhere
**J18.***	Pneumonia, unspecified organism
**J20.***	Acute bronchitis
**J21.***	Acute bronchiolitis
**J22**	Unspecified acute lower respiratory tract infection
**J40**	Bronchitis, not specified as acute or chronic
**J80**	Acute respiratory distress syndrome
**J98.8**	Other specified respiratory disorders
**A22.1**	Pulmonary anthrax
**A37.01**	Whooping cough: *Bordetella pertussis*, pneumonia
**A37.11**	Whooping cough: *B. parapertussis*, pneumonia
**A37.81**	Whooping cough: other *Bordetella*, pneumonia
**A37.91**	Whooping cough, unspecified species, pneumonia
**A48.1**	Legionnaire's Disease
**B25.0**	Cytomegaloviral pneumonitis
**B44.0**	Invasive pulmonary aspergillosis
**B97.4**	Respiratory syncytial virus as the cause of diseases classified elsewhere
**O98.5**	Other viral diseases complicating pregnancy, childbirth and the puerperium
**U07.1**	COVID-19, virus identified (confirmed diagnosis)
**U07.2**	COVID-19, virus not identified

*Includes all downstream codes beyond the decimal place

© 2024 Havers FP et al. *JAMA Network Open*

eTable 3. Estimated multipliers for under-detection of respiratory syncytial virus among hospitalized adults in RSV-NET, 2016–17 through 2022–23 seasons.

Season[a]	Age group (years)	Testing frequency (t)	Test sensitivity (s)[b]	Probability of detection (d=t*s)	Multiplier (m=1/d) (95% CI)	Unadjusted rate	Adjusted rate (95% CI)	Ratio of ICU admissions to hospitalizations	Adjusted ICU admission rate	Perc in-ho deat hosp ti
**2016–17**	18–49	30.4%	65.9%	20.1%	5.0 (3.3–9.8)	1.7	8.6 (5.7–16.8)	22.7%	1.9 (1.3–3.8)	2.
	50–64	33.1%	66.3%	21.9%	4.6 (3.3–7.6)	9.2	41.9 (29.9–69.5)	26.0%	10.9 (7.8–18.1)	4.
	65–74	31.5%	66.2%	20.9%	4.8 (3.4–8.3)	17.5	83.9 (58.8–146.1)	22.9%	19.2 (13.5–33.5)	3.
	≥75	27.7%	66.4%	18.4%	5.4 (3.6–10.8)	52.5	285.6 (190.8–568.1)	15.3%	43.8 (29.2–87.1)	6.
	All adults	30.0%	66.6%	20.0%	5 (3.5-9)	8.8	48.9 (33.4-91.5)	20.5%	10 (6.8-18.8)	4.
**2017–18**	18–49	37.4%	66.1%	24.7%	4.0 (3.1–5.9)	3.0	12 (9.2–17.4)	19.7%	2.4 (1.8–3.4)	2.
	50–64	39.8%	66.2%	26.4%	3.8 (2.9–5.6)	16.6	63.1 (47.5–93.9)	22.9%	14.4 (10.9–21.5)	3.
	65–74	34.3%	66.1%	22.7%	4.4 (3.2–7.3)	34.1	150.1 (107.5–248.6)	20.8%	31.2 (22.3–51.6)	4.
	≥75	35.5%	66.4%	23.6%	4.2 (3–7.2)	97.0	411.4 (292.1–695.4)	14.2%	58.6 (41.6–99)	5.
	All adults	36.2%	66.5%	24.1%	4.2 (3.2-5.9)	11.5	52.9 (40.4-76.8)	18.6%	9.8 (7.5-14.3)	3.
**2018–19**	18–49	37.1%	66.0%	24.5%	4.1 (3.2–5.7)	2.5	10.2 (8–14.2)	24.3%	2.5 (1.9–3.4)	1.
	50–64	37.3%	66.2%	24.7%	4.1 (3.1–5.8)	10.9	44 (33.8–62.9)	19.6%	8.6 (6.6–12.3)	2.
	65–74	36.5%	66.0%	24.1%	4.2 (3.2–6.1)	23.7	98.5 (75–143.3)	19.5%	19.2 (14.6–27.9)	4.
	≥75	35.1%	66.3%	23.2%	4.3 (3.3–6.3)	63.5	273.3 (206.7–403.2)	16.0%	43.7 (33–64.4)	3.
	All adults	36.2%	66.5%	24.1%	4.2 (3.2-5.9)	11.5	52.9 (40.4-76.8)	18.6%	9.8 (7.5-14.3)	3.
**2019–20**	18–49	42.4%	65.9%	27.9%	3.6 (3.2–4)	3.4	12 (10.9–13.5)	17.8%	2.1 (1.9–2.4)	0.
	50–64	47.2%	66.0%	31.2%	3.2 (2.7–3.9)	13.7	43.9 (37.6–52.8)	26.5%	11.6 (10–14)	3.
	65–74	46.9%	66.0%	31.0%	3.2 (2.8–3.8)	27.3	88.3 (77–103.5)	20.6%	18.2 (15.8–21.3)	3.
	≥75	44.4%	66.2%	29.4%	3.4 (2.9–4.1)	73.5	250.3 (213.9–301.7)	19.6%	49.1 (42–59.2)	6.
	All adults	44.5%	66.3%	29.5%	3.4 (3-3.9)	14.1	51.4 (44-60.8)	21.3%	11 (9.4-12.9)	4.
**2020–21**	18–49	28.7%	66.3%	19.0%	5.3 (4.1–7.2)	1.0	5.2 (4.1–7.2)	23.0%	1.2 (1–1.7)	2.
	50–64	30.0%	66.4%	19.9%	5.0 (3.9–7)	2.7	13.6 (10.7–18.9)	19.6%	2.7 (2.1–3.7)	2.
	65–74	31.8%	66.4%	21.1%	4.7 (3.7–6.6)	5.7	27 (21–37.5)	17.7%	4.8 (3.7–6.6)	5.
	≥75	31.7%	66.5%	21.1%	4.7 (3.7–6.7)	7.7	36.5 (28.3–51.3)	13.5%	4.9 (3.8–6.9)	6.
	All adults	30.2%	66.7%	20.2%	5 (3.9-6.8)	2.5	12.9 (10.5-17.9)	18.4%	2.4 (1.9-3.3)	4.
**2021–22**	18–49	43.2%	66.4%	28.6%	3.5 (2.9-4.4)	3.1	10.7 (8.9-13.4)	20.9%	2.2 (1.9-2.8)	2.

© 2024 Havers FP et al. *JAMA Network Open*

	50–64	49.0%	66.4%	32.5%	3.1 (2.5-4)	10.9	33.7 (27.5-43.4)	17.6%	5.9 (4.9-7.7)	4.
	65–74	50.7%	66.4%	33.7%	3 (2.5-3.7)	19.7	58.5 (48.5-73.6)	17.1%	10 (8.3-12.6)	3.
	≥75	49.0%	66.5%	32.6%	3.1 (2.5-3.9)	50.8	156.1 (128.4-199.1)	13.6%	21.3 (17.5-27.2)	5.
		47.6%	66.6%	31.7%	3.2 (2.6-4)	10.5	35.2 (28.6-44.9)	16.6%	5.8 (4.8-7.5)	4.
**2022–23**	18–49	56.1%	66.3%	37.2%	2.7 (2.3-3.3)	4.9	13.1 (11-16.1)	20.4%	2.7 (2.3-3.3)	3.
	50–64	61.2%	66.4%	40.6%	2.5 (2.1-3)	19.3	47.4 (40.6-57)	21.8%	10.4 (8.9-12.4)	2.
	65–74	62.3%	66.5%	41.4%	2.4 (2.1-2.9)	41.6	100.5 (85.4-121.9)	21.7%	21.8 (18.6-26.5)	4.
	≥75	61.6%	66.6%	41.0%	2.4 (2.1-3)	100.3	244.7 (207.9-297.3)	16.9%	41.4 (35.2-50.3)	6.
	All adults	60.7%	66.6%	40.4%	2.5 (2.1-3)	20.3	54.1 (46-65.6)	19.8%	10.7 (9.1-13)	4.

Abbreviations: CI: Confidence interval. ICU: Intensive care unit. [a] Season is defined as October–April for 2016-17 through 2019-20; for 2020-21 through 2022-23, season is defined as October - September. [b] Sensitivity of 66% and 29% were assumed for RT-PCR tests and antigen tests, respectively.

© 2024 Havers FP et al. *JAMA Network Open*

eTable 4. Estimated rates and number of respiratory syncytial virus hospitalizations in the U.S. by age group and season assuming a higher diagnostic test sensitivity (92%) to account for under-detection by nasopharyngeal swabs.

Age group (years)	Season[a]	Adjusted hospitalization rate	Rate lower confidence limit	Rate Upper confidence limit	Estimated Hospitalizations	Lower confidence limit	Upper confidence limit
**18–49 years**	2016–17	6.2	4.2	12.1	9,000	6,000	17,000
	2017–18	8.7	6.7	12.5	12,000	9,000	17,000
	2018–19	7.4	5.8	10.2	10,000	8,000	14,000
	2019–20	8.7	7.9	9.7	12,000	11,000	13,000
	2020–21	3.8	3	5.2	5,000	4,000	7,000
	2021–22	7.7	6.4	9.7	11,000	9,000	13,000
	2022–23	9.5	8	11.6	13,000	11,000	16,000
**50–64 years**	2016–17	30.4	21.8	50.1	19,000	14,000	32,000
	2017–18	45.7	34.5	67.6	29,000	22,000	43,000
	2018–19	31.9	24.6	45.4	20,000	15,000	29,000
	2019–20	31.9	27.4	38.2	20,000	17,000	24,000
	2020–21	9.9	7.7	13.7	6,000	5,000	9,000
	2021–22	24.4	19.9	31.3	16,000	13,000	20,000
	2022–23	34.3	29.4	41.1	22,000	19,000	26,000
**65–74 years**	2016–17	60.7	42.7	105.2	18,000	13,000	31,000
	2017–18	108.8	78.1	179.5	33,000	24,000	55,000
	2018–19	71.1	54.3	103.1	22,000	17,000	32,000
	2019–20	65.5	57.1	76.9	21,000	19,000	25,000
	2020–21	19.5	15.3	27.1	6,000	5,000	9,000
	2021–22	42.4	35.2	53.3	14,000	12,000	18,000
	2022–23	72.8	62	88.2	25,000	21,000	30,000
**≥75 years**	2016–17	206.9	138.3	410.4	44,000	29,000	87,000
	2017–18	298.2	211.9	503	65,000	46,000	110,000
	2018–19	198	149.9	291.5	45,000	34,000	66,000
	2019–20	181.5	155.4	218.1	42,000	36,000	50,000
	2020–21	26.4	20.5	37.1	6,000	5,000	9,000
	2021–22	113.1	93.1	144.1	25,000	21,000	32,000
	2022–23	177.5	150.9	215.4	43,000	36,000	52,000
**≥65 years**	122.2	82.8	232.5	62,000	42,000	118,000	122.2
	187.1	133.7	315.1	98,000	70,000	165,000	187.1
	123.9	94.3	181.3	67,000	51,000	98,000	123.9
	113.2	98.8	134.7	63,000	55,000	75,000	113.2
	21.6	18	32.3	12,000	10,000	18,000	21.6
	69.8	59.1	89.5	39,000	33,000	50,000	69.8
	117.7	98.6	141.9	68,000	57,000	82,000	117.7

© 2024 Havers FP et al. *JAMA Network Open*

**Overall**	35.8	24.7	66.4	90,000	62,000	167,000	35.8
	54.9	39.9	88.8	139,000	101,000	225,000	54.9
	38	29	55.3	97,000	74,000	141,000	38
	37	32.3	43.6	95,000	83,000	112,000	37
	9	7.4	13.2	23,000	19,000	34,000	9
	25.5	21.3	32.1	66,000	55,000	83,000	25.5
	39.5	33.4	47.5	103,000	87,000	124,000	39.5

Rates of RSV-associated hospitalizations were adjusted for under-detection of RSV infection due to testing practices and diagnostic test sensitivity. Sensitivity of 92% and 29% were assumed for RT-PCR tests and antigen tests, respectively. [a] Season is defined as October–April for 2016-17 through 2019-20; for 2020-21 through 2022-23, season is defined as October - September.

© 2024 Havers FP et al. *JAMA Network Open*

eTable 5. Adjusted[a] hospitalization rates, estimated hospitalizations, intensive care unit (ICU) admissions and in-hospital deaths with rates shown in 5-year age bands for adults aged ≥50 years and for all adults aged ≥60 and ≥65 years.

Age group (years)	Season[a]	Adjusted[b] hospitalization rate (95% Confidence Interval)	Estimated Hospitalizations (95% Confidence Interval)	Estimated ICU admissions (95% Confidence Interval)	Estimate d (95% Confi
**50–54**	2016–17	23.4 (17.5–35.1)	5,000 (4,000–7,000)	1,400 (1,200–2,000)	130 (
	2017–18	42.0 (31.4–63.5)	9,000 (7,000–13,000)	1,500 (1,200–2,200)	260 (
	2018–19	27.1 (20.8–38.8)	6,000 (4,000–8,000)	1,200 (800–1,600)	50
	2019–20	28.8 (24.1–35.9)	6,000 (5,000–7,000)	1,400 (1,200–1,700)	150 (
	2020–21	8.3 (6.6–11.2)	2,000 (1,000–2,000)	300 (100–300)	0
	2021–22	24.8 (20.3-31.8)	5,000 (4,000-7,000)	800 (700-1,200)	130 (
	2022–23	29.8 (25-36.8)	6,000 (5,000-8,000)	1,100 (900-1,500)	90 (
**55–59**	2016–17	46.3 (32–83.6)	10,000 (7,000–18,000)	2,300 (1,600–4,200)	260 (
	2017–18	58.8 (44.7–86)	13,000 (10,000–19,000)	2,800 (2,200–4,100)	320 (
	2018–19	47.3 (35.4–71.5)	10,000 (8,000–16,000)	2,000 (1,600–3,200)	300 (
	2019–20	40.5 (34.6–49)	9,000 (7,000–11,000)	2,400 (1,800–2,900)	290 (
	2020–21	13.5 (10.5–19)	3,000 (2,000–4,000)	700 (400–900)	170 (
	2021–22	32.3 (26.3-41.7)	7,000 (6,000-9,000)	1,100 (900-1,400)	280 (
	2022–23	49.2 (41.5-60.4)	10,000 (9,000-13,000)	2,400 (2,100-3,100)	370 (
**60–64**	2016–17	50.2 (35.3–86.8)	10,000 (7,000–17,000)	2,700 (1,900–4,600)	710 (5
	2017–18	90.1 (64–152.2)	18,000 (13,000–31,000)	4,900 (3,500–8,400)	820 (5
	2018–19	60.4 (45.2–91)	12,000 (9,000–19,000)	2,300 (1,700–3,600)	290 (
	2019–20	63.2 (53.2–77.8)	13,000 (11,000–16,000)	3,700 (3,100–4,500)	390 (
	2020–21	18.8 (13.6–30.2)	4,000 (3,000–6,000)	800 (600–1,200)	0
	2021–22	42.2 (33.6-56.5)	9,000 (7,000-12,000)	1,800 (1,400-2,400)	460 (
	2022–23	65.1 (54.2-81.4)	14,000 (11,000-17,000)	3,000 (2,400-3,700)	380 (
**65–69**	2016–17	76.4 (53.3–134.5)	13,000 (9,000–23,000)	3,100 (2,100–5,400)	690 (4
	2017–18	137 (95.3–244.1)	23,000 (16,000–42,000)	5,000 (3,500–9,200)	920 (6
	2018–19	85.6 (65.1–125)	15,000 (11,000–22,000)	3,200 (2,400–4,700)	700 (5
	2019–20	79.1 (66.9–96.7)	14,000 (12,000–17,000)	2,800 (2,400–3,300)	400 (
	2020–21	23.8 (18.4–33.6)	4,000 (3,000–6,000)	800 (600–1,200)	220 (
	2021–22	54.6 (44.3-70.9)	10,000 (8,000-13,000)	2,000 (1,600-2,700)	450 (
	2022–23	87.2 (73-108.1)	16,000 (14,000-20,000)	3,700 (3,300-4,700)	810 (7
**70–74**	2016–17	92.7 (63.6–170.9)	12,000 (8,000–22,000)	2,700 (1,800–4,900)	280 (
	2017–18	169.4 (122.3–275.2)	23,000 (16,000–37,000)	4,500 (3,200–7,300)	1,260 (
	2018–19	116.3 (87.3–174.2)	16,000 (12,000–24,000)	2,800 (2,100–4,200)	750 (5
	2019–20	98.7 (86.3–115.2)	14,000 (13,000–17,000)	3,000 (2,800–3,600)	620 (
	2020–21	29.9 (23.1–42.3)	4,000 (3,000–6,000)	600 (500–900)	210 (
	2021–22	63 (51.6-80.7)	10,000 (8,000-12,000)	1,400 (1,100-1,700)	220 (
	2022–23	116.9 (99.4-142)	18,000 (15,000-22,000)	3,700 (3,100-4,500)	780 (
**75–79**	2016–17	192.6 (124.5–425.7)	17,000 (11,000–37,000)	3,500 (2,300–7,700)	490 (3
	2017–18	263.8 (184.6–462.1)	24,000 (17,000–43,000)	5,100 (3,600–9,100)	1,660 (1

© 2024 Havers FP et al. *JAMA Network Open*

	2018–19	167 (124.8–252.3)	16,000 (12,000–24,000)	3,200 (2,400–4,900)	520 [illegible]
	2019–20	177.6 (147.4–223.4)	18,000 (15,000–22,000)	4,200 (3,500–5,100)	1,230 [illegible]
	2020–21	26.3 (20.5–36.5)	3,000 (2,000–4,000)	700 (400–900)	260 [illegible]
	2021–22	105.2 (88.4-129.9)	10,000 (9,000-13,000)	1,600 (1,500-2,100)	570 [illegible]
	2022–23	154.1 (129.9-189.3)	17,000 (14,000-21,000)	3,200 (2,600-3,900)	1,090 [illegible]
**≥80**	2016–17	348.1 (233.3–685.9)	43,000 (29,000–85,000)	5,700 (3,900–11,300)	3,140 [illegible]
	2017–18	514.6 (363.8–879.4)	65,000 (46,000–111,000)	7,700 (5,400–13,100)	3,030 [illegible]
	2018–19	355.7 (268.9–525)	46,000 (35,000–68,000)	6,600 (5,100–9,800)	1,910 [illegible]
	2019–20	308.7 (261.5–376.8)	41,000 (34,000–49,000)	7,500 (6,200–9,000)	2,790 [illegible]
	2020–21	43.4 (33.4–61.9)	6,000 (4,000–8,000)	600 (400–700)	270 [illegible]
	2021–22	195.3 (158.5-254.2)	24,000 (19,000-31,000)	3,000 (2,400-3,800)	1,150 [illegible]
	2022–23	319.8 (271.2-389.7)	42,000 (36,000-51,000)	6,800 (5,800-8,200)	2,870 [illegible]
**≥60**[c]	2016–17	134.4 (90.5-260.3)	95,000 (64,000-184,000)	17,700 (12,000-33,900)	5,310 [illegible]
	2017–18	210.5 (148.6-363.3)	153,000 (108,000-264,000)	27,200 (19,200-47,100)	7,690 [illegible]
	2018–19	140.7 (105.9-210.4)	105,000 (79,000-157,000)	18,100 (13,700-27,200)	4,170 [illegible]
	2019–20	130.8 (111.2-158.3)	100,000 (85,000-121,000)	21,200 (18,000-25,500)	5,430 [illegible]
	2020–21	27.5 (19.6-39.2)	21,000 (15,000-30,000)	3,500 (2,500-4,900)	960 [illegible]
	2021–22	81.7 (66.2-105.1)	63,000 (51,000-81,000)	9,800 (8,000-12,700)	2,850 [illegible]
	2022–23	135.6 (114-166)	107,000 (90,000-131,000)	20,400 (17,200-25,000)	5,930 [illegible]
**≥65**	2016–17	167.5 (112.3–321.2)	85,000 (57,000–163,000)	14,900 (10,000–28,200)	4,630 [illegible]
	2017–18	259.7 (185.2–435.4)	136,000 (97,000–228,000)	22,400 (16,000–37,400)	6,910 [illegible]
	2018–19	172 (131.3–251.6)	93,000 (71,000–136,000)	15,900 (12,200–23,300)	3,870 [illegible]
	2019–20	156.3 (133–186.9)	87,000 (74,000–104,000)	17,400 (14,700–20,700)	5,010 [illegible]
	2020–21	30.5 (25.2–43.1)	17,000 (14,000–24,000)	2,700 (2,100–3,700)	970 [illegible]
	2021–22	98.5 (78.8-123.5)	55,000 (44,000-69,000)	8,200 (6,500-10,300)	2,450 [illegible]
	2022–23	160.9 (136.7-193.8)	93,000 (79,000-112,000)	17,400 (14,800-20,900)	5,550 [illegible]

Abbreviations: ICU: Intensive care unit. [a] Season is defined as October–April for 2016-17 through 2019-20; for 2020-21 through 2022-23, season is defined as October - September. [b] Rates of RSV-associated hospitalizations were adjusted for under-detection of RSV infection due to testing practices and diagnostic test sensitivity. [c] Estimates for adults aged ≥60 years are shown separately to reflect potential vaccine-avertable RSV hospitalizations, ICU admissions, and in-hospital deaths; as of June 2024, RSV vaccination is recommended for all adults aged ≥75 years and those aged 60–74 years who are at increased risk of severe RSV disease.

© 2024 Havers FP et al. *JAMA Network Open*

eFigure. Proportion of adults hospitalized with respiratory syncytial virus by age group and season.[a]

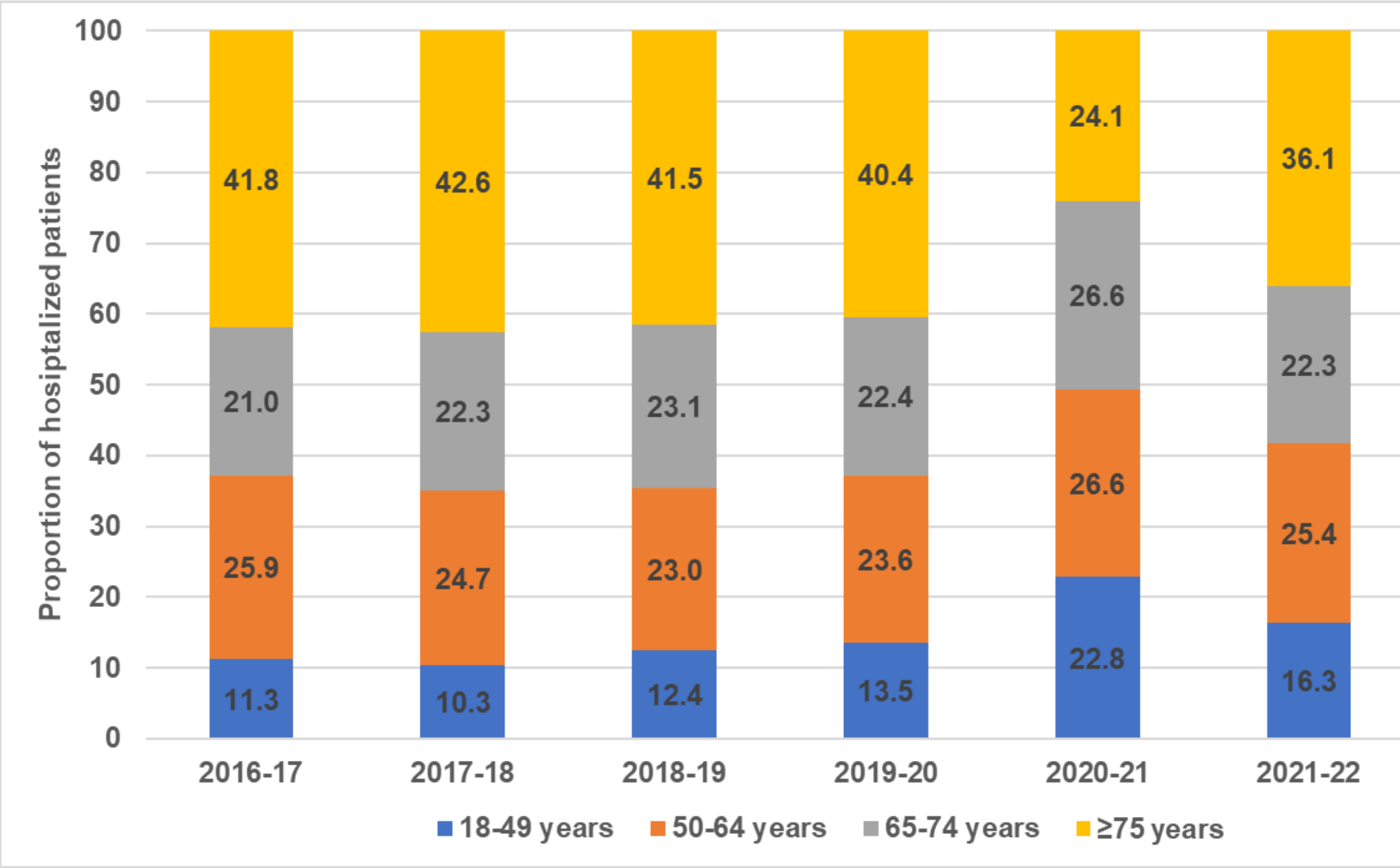

[a] Season is defined as October–April for 2016-17 through 2019-20; for 2020-21 through 2022-23, season is defined as October - September.

© 2024 Havers FP et al. *JAMA Network Open*

eMethods.

The Respiratory Virus Hospitalizations Surveillance Network comprises three networks that conduct population-based surveillance for laboratory-confirmed hospitalizations associated with influenza, respiratory syncytial virus (RSV), and COVID-19. The surveillance platforms, known as COVID-NET, RSV-NET and FluSurv-NET have similar catchment areas and use similar methods to identify patients and collect data. RSV-NET uses burden methodology developed by the Influenza Division at CDC to estimate influenza hospitalizations using FluSURV-NET data, as described in detail by Reed, et al, and others.[1,2] These methods, as applied to RSV-NET are summarized below, including RSV-NET specific methodological adjustments.

Note that prior to 2020, surveillance was conducted between October – April; this period was defined as the “season” for 2016-17 through 2019-20 and October – September for 2020-21 through 2022-23. Prior to 2020, almost all RSV hospitalizations likely occurred between October – April; seasonal estimates for years in which surveillance was conducted in October – April only were assumed to approximate annual estimates.

Adjustment for sensitivity

To correct for under-detection of RSV hospitalizations, we adjusted the reported rate of laboratory-confirmed RSV hospitalizations for each age group by the proportion of patients with ARI tested for RSV (see below) and the average sensitivity of RSV testing. Based on systematic reviews, we presumed an initial sensitivity of 92%[3] for multiplex RT-PCR (compared with the gold standard singleplex RT-PCR) and 29% for rapid antigen assays.[4] There is an increasing body of literature that indicates that nasopharyngeal (NP) PCR testing, by far the most commonly used test among adult hospitalized patients in RSV-NET, might be insensitive for identifying RSV in patients who are hospitalized with lower respiratory tract disease. In a study of patients hospitalized with laboratory-confirmed pneumonia with

© 2024 Havers FP et al. *JAMA Network Open*

PCR testing for multiple pathogens, acute and convalescent serology testing increased the number of patients who likely had RSV as the etiology of their pneumonia by 40%, although the absolute numbers of identified patients were small.[5] Another study showed that among patients who had RSV detected on bronchoalveolar lavage (BAL), only 61.5% also had RSV on NP swab; RSV was the most discordant between BAL and NP results of all the pathogens studied.[6] In a systematic review and meta-analysis examining under-detection of RSV in adults due to diagnostic testing limitations, the authors analyzed data from 154 studies and found that while generally RT-PCR is the most sensitive modality for testing, adding specimen types such as paired serology and sputum to NP or nasal swab RT-PCR increased RSV detection, on average, by 50% to 66%, respectively.[3] Based on these results and similar to what was used in an industry-sponsored meta-analysis and systematic review, based on seven studies demonstrating under-detection,[7] we incorporated an additional multiplier of 1.5 to adjust for lack of sensitivity of clinical tests of a single type.

Adjustment for undertesting

Because RSV testing in RSV-NET areas is performed at the discretion of the healthcare provider, a person with RSV is only identified if the patient is tested for RSV and the test correctly identifies RSV infection. Patients with RSV are missed if testing is not performed or if the tests are not perfectly sensitive. To adjust for under-detection of RSV–associated hospitalizations, additional data were collected to estimate: 1) the proportion of patients hospitalized with acute respiratory infections (ARI) who would have been tested for RSV and 2) the probability that a person who truly had RSV would test positive for RSV. This analysis was reviewed by the US Centers for Disease Control and Prevention (CDC), deemed not research, and was conducted consistent with applicable federal law and CDC policy.[1]

---

[1] See e.g., 45 C.F.R. part 46.102(l)(2), 21 C.F.R. part 56; 42 U.S.C. §241(d); 5 U.S.C. §552a; 44 U.S.C. §3501 et seq.

To identify eligible persons hospitalized with ARI, all sites selected a convenience sample of hospitals in their catchment area. These hospitals identified all patients who had been admitted with respiratory infections using a discharge audit of respiratory illness related ICD-10 codes (see supplementary table 1). A stratified random sample of eligible patients per month by age group (18–49, 50–64, ≥65 years) were selected; laboratory records or medical charts were reviewed to identify if patients were tested for RSV, and, if so, what type of test was used. Data were analyzed within age groups.

The overall level of under-detection was summarized using a multiplier that represents the expect number of true RSV hospitalizations per reported hospitalization. This was calculated as:

Multiplier = 1/ (Frequency of RSV testing x sensitivity of RSV test type) * 1.5 adjustment

eReferences

1. Reed C, Chaves SS, Daily Kirley P, et al. Estimating influenza disease burden from population-based surveillance data in the United States. *PloS one*. 2015;10(3):e0118369. doi:10.1371/journal.pone.0118369

2. Rolfes MA, Foppa IM, Garg S, et al. Annual estimates of the burden of seasonal influenza in the United States: A tool for strengthening influenza surveillance and preparedness. *Influenza Other Respir Viruses*. Jan 2018;12(1):132-137. doi:10.1111/irv.12486

3. Onwuchekwa C, Moreo LM, Menon S, et al. Under-ascertainment of Respiratory Syncytial Virus infection in adults due to diagnostic testing limitations: A systematic literature review and meta-analysis. *The Journal of infectious diseases*. Jan 20 2023;doi:10.1093/infdis/jiad012

4. Chartrand C, Tremblay N, Renaud C, Papenburg J. Diagnostic Accuracy of Rapid Antigen Detection Tests for Respiratory Syncytial Virus Infection: Systematic Review and Meta-analysis. *Journal of clinical microbiology*. Dec 2015;53(12):3738-49. doi:10.1128/JCM.01816-15

© 2024 Havers FP et al. *JAMA Network Open*

5. Zhang Y, Sakthivel SK, Bramley A, et al. Serology Enhances Molecular Diagnosis of Respiratory Virus Infections Other than Influenza in Children and Adults Hospitalized with Community-Acquired Pneumonia. *Journal of clinical microbiology*. Oct 19 2016;doi:10.1128/JCM.01701-16

6. Bouzid D, Le Hingrat Q, Salipante F, et al. Agreement of respiratory viruses' detection between nasopharyngeal swab and bronchoalveolar lavage in adults admitted for pneumonia: a retrospective study. *Clinical microbiology and infection : the official publication of the European Society of Clinical Microbiology and Infectious Diseases*. Jan 25 2023;doi:10.1016/j.cmi.2022.12.024

7. McLaughlin JM, Khan F, Begier E, Swerdlow DL, Jodar L, Falsey AR. Rates of Medically Attended RSV Among US Adults: A Systematic Review and Meta-analysis. *Open forum infectious diseases*. Jul 2022;9(7):ofac300. doi:10.1093/ofid/ofac300

© 2024 Havers FP et al. *JAMA Network Open*
