# Supplementary material for: Burden of Respiratory Syncytial Virus–Associated Hospitalizations in US Adults, October 2016 to September 2023
Source: JAMA Netw Open. 2024 Nov 13;7(11):e2444756. doi: 10.1001/jamanetworkopen.2024.44756 (PMC11561688; doi:10.1001/jamanetworkopen.2024.44756)
Supplement: Supplement 2. — Data Sharing Statement [file jamanetwopen-e2444756-s002.pdf]

Data Sharing Statement

Havers. Burden of Respiratory Syncytial Virus–Associated Hospitalizations in US Adults, October 2016 to September 2023. *JAMA Netw Open*. Published November 13, 2024. doi:10.1001/jamanetworkopen.2024.44756

Data
Data available: No
